# Neuroimaging in schizophrenia: an overview of findings and their implications for synaptic changes

**DOI:** 10.1038/s41386-022-01426-x

**Published:** 2022-09-02

**Authors:** Oliver D. Howes, Connor Cummings, George E. Chapman, Ekaterina Shatalina

**Affiliations:** 1 Psychiatric Imaging Group, MRC London Institute of Medical Sciences, Hammersmith Hospital, Imperial College London, London, UK; 2 Department of Psychosis Studies, Institute of Psychiatry, Psychology & Neuroscience, King’s College London, London, UK; 3 South London and Maudsley NHS Foundation Trust, London, UK; 4 Clare Hall (College), University of Cambridge, Cambridge, UK

**Keywords:** Schizophrenia, Schizophrenia, Psychosis

## Abstract

Over the last five decades, a large body of evidence has accrued for structural and metabolic brain alterations in schizophrenia. Here we provide an overview of these findings, focusing on measures that have traditionally been thought to reflect synaptic spine density or synaptic activity and that are relevant for understanding if there is lower synaptic density in the disorder. We conducted literature searches to identify meta-analyses or other relevant studies in patients with chronic or first-episode schizophrenia, or in people at high genetic or clinical risk for psychosis. We identified 18 meta-analyses including over 50,000 subjects in total, covering: structural MRI measures of gyrification index, grey matter volume, grey matter density and cortical thickness, neurite orientation dispersion and density imaging, PET imaging of regional glucose metabolism and magnetic resonance spectroscopy measures of N-acetylaspartate. We also review preclinical evidence on the relationship between ex vivo synaptic measures and structural MRI imaging, and PET imaging of synaptic protein 2A (SV2A). These studies show that schizophrenia is associated with lower grey matter volumes and cortical thickness, accelerated grey matter loss over time, abnormal gyrification patterns, and lower regional SV2A levels and metabolic markers in comparison to controls (effect sizes from ~ −0.11 to −1.0). Key regions affected include frontal, anterior cingulate and temporal cortices and the hippocampi. We identify several limitations for the interpretation of these findings in terms of understanding synaptic alterations. Nevertheless, taken with post-mortem findings, they suggest that schizophrenia is associated with lower synaptic density in some brain regions. However, there are several gaps in evidence, in particular whether SV2A findings generalise to other cohorts.

## Introduction

Schizophrenia is generally a chronic and disabling mental health condition associated with positive (psychotic symptoms such as delusions, hallucinations), negative (such as anhedonia, asociality) and cognitive symptoms (such as impaired working memory and executive function) [1]. The first positive symptoms typically develop after cognitive and negative symptoms [1].

Excessive loss of synapses has been hypothesised to lead to the disorder, and may also occur during the disorder, contributing to long-term disability [2–4]. It has the potential to explain a number of aspects of the pathophysiology, epidemiology and clinical presentation of the disorder [5].

There are two key bodies of in vitro evidence supporting the hypothesis that there is lower synaptic density in schizophrenia. Firstly, post-mortem studies of brain samples from patients with schizophrenia have been used to investigate synaptic markers. This includes measuring protein and mRNA levels of synaptophysin, a presynaptic protein considered the in vitro gold standard marker for synaptic density [6]. Upon meta-analysis, synaptophysin levels have been shown to be significantly lower in the cingulate cortex (predominantly anterior part), frontal cortex and hippocampi of patients relative to controls [6]. In addition to this, markers of postsynaptic density are also lower across a number of brain regions in patients with schizophrenia relative to control samples [7]. Studies that use electron microscopy and manual counting also show lower synaptic spine levels in cortical regions [8–10].

Secondly, studies of neurones cultured from induced pluripotent stem cells (iPSCs) from patients with schizophrenia have investigated synaptic markers, generally, relative to non-isogenic control lines. These studies show impaired branching and synaptic formation [11, 12], as well as providing evidence of excessive elimination of synapses in vitro associated with schizophrenia genotypes [13, 14].

However, whilst these findings provide evidence for lower synaptic markers in schizophrenia, it is not clear if this is the case in vivo. To address this, we aim to review and critique in vivo neuroimaging findings that potentially capture information about synaptic density or synaptic activity to consider the nature of the brain changes in schizophrenia and address the question of whether there is lower synaptic density in schizophrenia. We consider structural imaging approaches using magnetic resonance imaging (MRI) measures of gyrification index, grey matter volume, grey matter density and cortical thickness, because changes in these have been interpreted as reflecting synaptic loss in schizophrenia [15–18]. We first focus on findings in patients with chronic schizophrenia, then in first episode psychosis and genetically and clinically high-risk individuals, and review longitudinal data where available. We then overview findings from positron emission tomography (PET) imaging of regional glucose metabolism, and magnetic resonance spectroscopy (MRS) measures of neuronal metabolic function in patients with schizophrenia. Finally, we review PET imaging of synaptic protein 2 A in schizophrenia, outline remaining questions and identify areas for future research.

## Methods

**Fig. 1:**
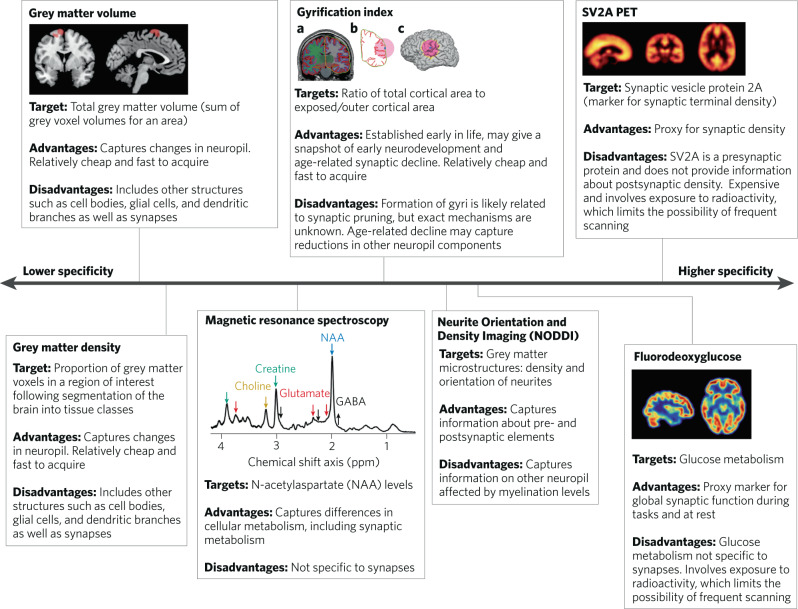
Overview of in vivo neuroimaging methods, summarising the main target and advantages and disadvantages in terms of capturing information related to synapses. The specificity line is not linear but illustrates the rank order of specificity for synapses. PET positron emission tomography, SV2A synaptic vesicle protein 2 A.

We conducted a series of literature searches to identify relevant studies using PubMed and Google Scholar databases (search from 1st January 1966 to 31st March 2022) and supplemented these with hand-searching of reference lists. We identified the most recent meta-analyses and summarise these to provide an overview of the magnitude and consistency of findings for each imaging approach. A summary of the imaging techniques included can be seen in Fig. 1. Where meta-analyses were not available, we identified the most recent systematic reviews or, where these were not available, the largest studies available to date. The search terms and numbers of key publications included can be found in the supplementary materials. Effect sizes throughout are defined as small if Hedges' *g*/Cohen’s *d* < |0.2 |, medium effect sizes include Hedges' *g*/Cohen’s *d* in the |0.2 |‒|0.5| range, and large effect sizes are defined as Hedges' *g*/Cohen’s *d* > |0.5|.

### Gyrification and gyrification index

Gyrification is the folding of the cerebral cortex which gives rise to characteristic peaks (gyri) and troughs (sulci). Gyrification increases the brain’s surface area:volume ratio, maximising the number of neurones and the efficiency of their communication [19]. The gyrification index (GI) is the ratio of the length of the complete (i.e. folded) cortical contour to the length of the outer (i.e. smooth) brain surface. Thus, a higher GI indicates more extensive cortical folding [20].

The only meta-analysis of GI studies to date found a lower local GI in schizophrenia patients than controls in fronto-temporal regions with a small effect size (Hedges’ *g* = −0.2) [21]. However, it included data from just six cross-sectional patient cohorts, partly because it only included studies published between 2010 and 2020, and it should be recognised that studies not included have found evidence for higher GI in schizophrenia [22, 23]. These inconsistencies may be at least partly explained by the stage of illness, as studies in younger patients (typically in their first episode) and those at high risk of psychosis predominantly show evidence for higher gyrification in the frontal cortex [22–27], whilst studies of patients with chronic illness tend to show lower gyrification relative to controls [27, 28].

In addition, longitudinal studies have shown that GI reduces with duration of psychosis in the same subjects [29, 30]. Thus, higher gyrification early in schizophrenia, followed by a process leading to reductions in GI in chronic illness, could explain the apparently conflicting findings between studies. Indeed, this idea is supported by a recent mega-analysis of data from over 670 individuals [27].

How might GI changes reflect synaptic changes? Synaptic density reflects the balance between synaptogenesis and synapse loss and is additionally affected by synaptic spine dynamics such as synaptic remodelling and stabilisation. Synaptogenesis increases rapidly early in neurodevelopment, exceeding the rate of synaptic loss, so that synaptic density increases markedly post-partum until it starts to level-off in early childhood [31–33]. Following this period, synaptic pruning is thought to exceed synaptogenesis, so that synaptic density declines into the third decade of life before plateauing from the fourth decade until older age. This process varies by brain region, with higher-order regions such as the prefrontal cortex maturing later [31–34]. Two large studies of over 1100 healthy individuals aged 3–83 years found that GI was highest at around 3 years and gradually declined throughout adulthood, similar to the trajectory of synaptic changes [35, 36]. Synaptic pruning may release tethering connections between sulci and gyri, causing sulcal widening and gyral peaking [19]. Supporting this, computational work shows that cortical folding in primates is determined through mechanical forces arising from synaptic connections between migrating axons [37]. These ‘tension-based’ mechanisms are considered key to cortical folding during neurodevelopment [38]. Importantly, this process could be exaggerated in schizophrenia, which may explain the longitudinal findings of GI reductions as the disease progresses [30].

However, there are two key limitations of GI measures and their application to the synaptic hypothesis. Firstly, there are inconsistent findings, particularly regarding whether first episode patients show higher or lower gyrification relative to controls, indicating that further work, particularly longitudinal studies in first episode patients, is needed. Secondly, GI is not a direct measure of synaptic density. Neuronal loss and demyelination, which are uncontrolled in most in vivo studies of gyrification [39], may contribute to altered GI [31, 40]. Moreover, the relationship between changes in GI and synaptic markers (such as synaptophysin or SV2A) remains unprobed. Thus, although GI differences in schizophrenia are consistent with synaptic alterations, they may not be due to synaptic alterations and further research is needed to establish the exact relationship between synaptic loss and GI changes.

### Grey matter volumes and cortical thickness

#### Studies of schizophrenia groups

Over the last three decades there have been more than 500 structural MRI studies including over 38,000 volunteers comparing brain volumes between people with schizophrenia and healthy controls [41–49]. Fortunately, these findings have been synthesised in a series of comprehensive meta-analyses. Comparisons between groups can be made between volumes of defined regions of interest, generally based on anatomically defined structures, or at the level of the voxel. These provide complementary information, with the former allowing hypotheses concerning particular anatomical structures to be tested, whilst the latter are not generally constrained by anatomical delineations and so may detect the pattern of differences across the whole brain and within regions.

In 2019, Kuo & Pogue-Geile undertook a meta-analysis comparing brain volumes between people with chronic schizophrenia and healthy controls [41]. Their results, summarised in Table 1 below, demonstrate statistically significant increases in lateral and third ventricle volumes, and significantly lower intracranial, total brain and total grey matter volumes, as well as smaller volumes of a number of grey matter brain structures, including bilateral frontal lobes, bilateral prefrontal lobes, bilateral superior temporal gyri, bilateral hippocampi, bilateral fusiform and left insula in schizophrenia [41]. The increases in ventricular volume are generally assumed to reflect loss of volume in grey matter, and potentially other structures, given evidence that intra-cranial volume is, if anything, smaller in people with schizophrenia [50].

**Table 1:** Summary of structural MRI findings by brain region for grey matter volumes in schizophrenia/people at risk of schizophrenia relative to healthy controls unless otherwise stated from recent meta-analyses, giving effect sizes (- indicates lower in schizophrenia).

_ROI_	First-degree relatives: 18 studies, *n* = 1228 first-degree relatives of people with schizophrenia including 49 monozygotic co-twins, 62 dizygotic co-twins, 171 offspring, 842 siblings, 104 parents (de Zwarte et al., 2019)		22q11.2 deletion syndrome: 24 studies, *n* = 998 22q11.2DS, 873 controls (Rogdaki et al., 2020)		Clinical high-risk with transition to psychosis (relative to clinical high-risk without transition): 28 studies, *n* = 1248 clinical high-risk, 1122 controls (Fortea et al., 2021)		First episode psychosis/early course schizophrenia, *n* = 3901 patients, 4040 controls, largely antipsychotic naïve/free (Brugger & Howes, 2017)		Chronic schizophrenia: 246 studies, *n* = 2830 patients, mean illness duration of 131 months, *n* = 3046 controls (Kuo & Pogue-Geile, 2019)	
	Mean effect size (*d*) corrected for ICV	*p*	Mean effect size (*g*)	*p*	Mean effect size (*g*)	*p*	Mean effect size (*g*)	*p*	Mean effect size (*d*)	*p*
Accumbens	−0.05	Not significant [95% CI: –0.13, 0.02]								
Amygdala	0.00	Not significant [95% CI: –0.11, 0.11]					−0.46	<0.001		
Anterior cingulate cortex (left)							−0.26	0.006		
Anterior cingulate cortex (right)					−0.39	0.0005				
Caudate	−0.03	Not significant [95% CI: –0.11, 0.04]					−0.11	Not significant (*p* = 0.23)		
Frontal lobe			−0.47	<0.001			−0.31	<0.001	−0.31	0.003
Fusiform (left)									−0.60	<0.005
Fusiform (right)									−0.45	
Grey matter (total)			−0.81	<0.001					−0.38	<0.001
Grey matter (cortical)	−0.11	*q* < 0.05 corrected								
Hippocampus	−0.06	Not significant [–0.14, 0.02]					−0.66	<0.001	−0.49	<0.001
Insula (left)									−0.46	0.045
Insula (right)									Not significant	
Intracranial volume	n/a								−0.12	<0.001
Lateral ventricles	0.09	*p* < 0.05 uncorrected					0.40	<0.001	0.44	<0.001
Prefrontal lobe									−0.40	<0.001
Pallidum	−0.05	Not significant [95% CI: –0.12, 0.02]								
Putamen	−0.07	Not significant [95% CI: –0.14, 0.01]					−0.31	0.11		
Superior temporal gyrus (left)									−0.57	0.01
Superior temporal gyrus (right)					−0.38	0.0008			−0.90	
Temporal lobe			−0.84	<0.001			−0.22	0.001	−1.09	0.029
Thalamus	−0.13	*q* < 0.05 corrected					−0.36	0.001		
Third ventricle	0.15	*q* < 0.05 corrected					0.43	<0.001	0.52	<0.001
Total brain volume			−0.96	<0.001					−0.25	<0.001

Dark grey boxes indicate data not reported, light grey boxes indicate no significant difference, and white boxes show significant differences.

*ICV* intracranial volume, *GMV* grey matter volume, *ROI* region of interest.

These results are consistent with those from a voxel-wise, coordinate-based meta-analysis by Glahn et al. [45], which included data from 1195 schizophrenia patients and 1262 healthy control subjects. They found lower grey matter density relative to controls in a network of regions, which included the bilateral insular cortex, anterior cingulate gyrus, left parahippocampal gyrus, middle frontal gyrus, postcentral gyrus and the thalamus [45]. Interestingly, data from 15 of the 31 studies analysed demonstrated increased grey matter density in some brain areas, notably striatal regions such as the left and right putamen and the right head of the caudate. As many of the patients in these studies had taken antipsychotic treatment for many years, and given that all antipsychotic drugs bind to D_2_ receptors, which are highly expressed in the striatum [51, 52], this could be an effect of antipsychotic treatment.

To exclude potentially confounding effects of antipsychotic use on grey matter morphology in schizophrenia, Gao et al. compiled evidence from 15 structural MRI studies comprising 486 drug-free patients and 485 healthy controls in 2018 [44]. In this analysis, researchers observed significantly lower grey matter volumes in four main neuroanatomical regions in patients with schizophrenia versus controls: left fusiform gyrus, left inferior frontal gyrus, right superior temporal gyrus and left supramarginal gyrus. These regions overlap with the regions identified in the other meta-analyses that included antipsychotic treated patients. Interestingly, this antipsychotic-free schizophrenia group did not show increased grey matter volume in the striatum but did show increases in right lingual gyrus and right superior frontal gyrus (orbital part) volumes. However, it should be noted that, antipsychotic-free subjects included in this study were not necessarily antipsychotic-naïve, as some patients had been treated but had undergone a medication wash-out prior to scanning, so it remains possible that prior treatment has had some influence on findings [44]. Notwithstanding this, the predominant findings are of decreased grey matter volumes in cortical and subcortical regions in schizophrenia, in conjunction with smaller areas of modest increases in grey matter volumes.

**Fig. 2:**
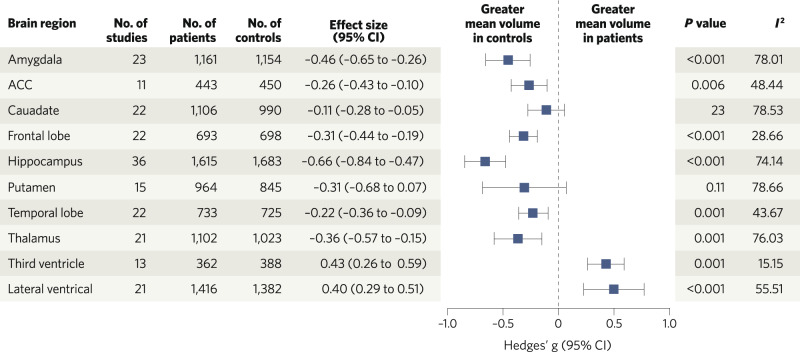
Brain volume differences in patients with first episode psychosis relative to controls. Meta-analytic results of regional brain volume differences between patients with first episode psychosis and healthy control subjects, adapted with permission from Brugger and Howes (2017). ACC anterior cingulate cortex.

These studies were predominantly in patients with chronic illnesses. This raises the question of whether grey matter volume alterations are present early in the course of illness or only emerge later. Findings by Brugger and Howes show statistically significant higher ventricular volumes and lower mean volumes of the amygdala, anterior cingulate cortex, frontal lobe, hippocampus, temporal lobe and thalamus in first episode psychosis when compared to controls (see Table 1 and Fig. 2 for details) [42]. Overall, the findings indicate reduced grey matter volume across several regions, particularly frontal and temporal cortical regions, in schizophrenia, and that these are also evident in the first episode psychosis subgroup, and after accounting for confounding factors such as the effects of long-term antipsychotic use. This raises the question of whether alterations are also seen in people at risk for schizophrenia. To address this, we now consider brain volume abnormalities observed in genetically and clinically high-risk populations.

#### Studies of people at familial and genetic high-risk of schizophrenia

The first-degree relatives of people with schizophrenia may carry genetic risk variants associated with schizophrenia. They are also likely to have shared similar environments. In 2019, de Zwarte et al. [53] showed that, after correction for intracranial volume, first-degree relatives of patients with schizophrenia had significantly smaller total brain (Cohen’s *d* = −0.16, *q* < 0.05, corrected), total cortical grey matter (*d* = −0.11, *q* < 0.05, corrected), total cerebral white matter (*d* = −0.12, *q* < 0.05, corrected) and cerebellar grey and white matter (both *d* = −0.09, *q* < 0.05, corrected) volumes relative to controls. The only subcortical subregion to demonstrate statistically significant lower grey matter volume relative to controls was the thalamus (*d* = −0.13, *q* < 0.05, corrected). No other regions of interest demonstrated statistically significant differences in volumes in this population when compared with healthy controls, including regions where alterations were found in the meta-analyses in schizophrenia. It should be noted that although de Zwarte et al. measured overall cortical grey matter, they did not investigate cortical subregions [53].

In 2020, Saarinen et al. also investigated volumetric differences in first-degree relatives of people with schizophrenia relative to controls but used a voxel-wise rather than region of interest approach. It had a smaller overall sample size (885 first-degree relatives versus 775 healthy controls) than the de Zwarte et al. meta-analysis [53, 54]. In contrast to the results of de Zwarte et al. above, this voxel-based study found no difference in grey matter volumes analysed at the voxel-level between first-degree relatives and controls. However, these analyses may be underpowered relative to the de Zwarte et al. analyses given the need to adjust for many more degrees of freedom in voxel-wise analyses and the smaller sample size than the de Zwarte et al. analysis.

Another approach, which addresses the issue that relatives may not carry genetic variants linked to schizophrenia, is to study individuals who are carriers of established genetic risk variants associated with schizophrenia, such as a genetic deletion at 22q11.2. In 2020, Rogdaki et al. published a meta-analysis of brain volume abnormalities in carriers of the 22q11.2 deletion presenting the with deletion syndrome (DS) [55]. Their results demonstrated significantly lower mean fronto-temporal volumes in 22q11.2DS individuals relative to controls (see Table 1). However, the 22q11.2DS population in this meta-analysis includes subjects with schizophrenia and comorbid neuropsychiatric illness(es), which means illness effects may contribute to findings [55].

In conclusion, the meta-analyses to date of those at familial and/or genetic risk of schizophrenia demonstrate lower grey matter volumes across a number of measures, including overall cortical grey matter, but not at the voxel level, and effect sizes are generally smaller than in schizophrenia. This indicates that whilst a component of the lower grey matter volumes seen in schizophrenia is heritable, there are grey matter differences that are likely specific to the disorder in degree.

#### Studies of people at clinical high-risk of schizophrenia

Clinical high risk (CHR) refers to individuals meeting clinical criteria, such as subthreshold positive psychotic symptoms and functional impairment, which mean they are at markedly increased risk of developing schizophrenia within a few years [56].

In 2021, Fortea at al. published a meta-analysis studying cortical grey matter in people meeting clinical high-risk criteria using a novel analytical method that combined grey matter volume measures with surface-based (cortical thickness) morphometry. This allowed them to synthesise findings from volumetric and surface-based morphometric studies by making use of t-maps and peak coordinates [57]. They found no significant differences in the combined grey matter volumetric/cortical thickness measures [43]. With respect to subcortical volumes, a large analysis by the ENIGMA Working Group in 2021, which compared data from 1792 individuals at CHR against 1377 healthy controls, also found no difference in subcortical volumes between groups [58]. Interestingly, however, statistically significant grey matter differences were seen within the CHR population. In the meta-analysis by Fortea et al., CHR individuals who subsequently transitioned to a psychotic disorder, predominantly schizophrenia, displayed lower calculated cortical grey matter (based on their combined volumetric/surfaced based thickness measures, as discussed above) in the right temporal lobe (*g* = −0.38) and anterior cingulate/paracingulate cortex (*g* = −0.39) relative to CHR participants who did not develop a disorder during the studies (Table 1) [43]. This suggests that lower grey matter volumes in fronto-temporal regions are associated with the prodrome to schizophrenia rather than the expression of sub-clinical symptoms, indicating specificity to the development of the disorder.

#### Longitudinal structural imaging studies

Given that, in absolute terms, the effect sizes for lower grey matter volumes are larger in people with chronic schizophrenia than in first episode samples (Table 1), a key question, then, is whether structural brain changes are progressive. However, there are other potential explanations, such as cohort effects. For example, first episode cohorts typically include a proportion of patients who recover and stay well, and are thus not included in samples of patients with chronic illness [59, 60]. It is possible that this subgroup has less marked grey matter alterations, although this remains to be tested. However, if this is the case, the failure to include the sub-group with a good outcome in studies of patients with chronic illness may explain the larger grey matter changes relative to controls than are seen in first episode studies. Longitudinal studies have addressed this issue by measuring changes in grey matter volumes over time in the same subjects.

**Fig. 3:**
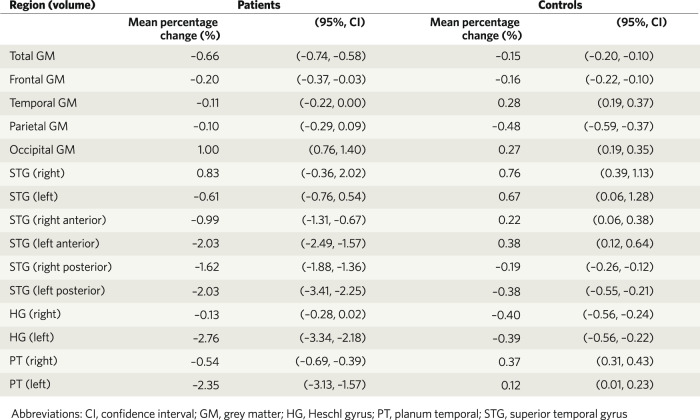
Estimated percentage change in regional grey matter volumes per year in patients with schizophrenia versus healthy controls in a meta-analysis of 19 longitudinal studies, including data from 813 patients and 718 controls. Copied with permission from Vita et al., 2012.

In 2012, Vita et al. meta-analysed data from 813 subjects with schizophrenia and 718 healthy controls. They found that the schizophrenia group demonstrated a statistically significant greater volume loss over time in total cortical grey matter and in other brain regions, including the left superior temporal gyrus, left anterior superior temporal gyrus; left Heschl gyrus; left planum temporale; and posterior superior temporal gyrus bilaterally [61]. As can be seen in Fig. 3, these differences are subtle, with about a 0.5% greater loss of total grey matter each year in the schizophrenia group than healthy controls.

These studies thus indicate that there is greater reduction in grey matter volumes in schizophrenia over time than in controls, but, as they predominantly included patients with chronic illness, it was unclear if this was present from illness onset. This was addressed in a systematic review by Gallardo-Ruiz et al. that focussed on studies of first episode patients. This found evidence for progressive grey matter volume loss in frontal, temporal (especially superior temporal regions), parietal cortices and a number of subcortical areas in patients from their first episode psychosis [62]. Similarly, further temporal analysis by Vita at al. in 2012 revealed that grey matter volume loss was most pronounced in the first episode psychosis subgroup with significant reductions observed in frontal, temporal and parietal lobes, and the left Heschl gyrus, compared to healthy controls. With respect to the clinical high-risk population, a 2021 systematic review by Merritt et al. echoed these findings, demonstrating that those suffering from psychotic experiences demonstrated a progressive decline in regional grey matter volumes, with changes most marked in the temporal, frontal, cingulate and parietal cortices [63].

#### Antipsychotic use and volumetric findings

Meta-analyses have shown that greater antipsychotic exposure is associated with greater case-control differences in both cross-sectional and longitudinal studies ([46–49] see supplement for discussion). However, findings from longitudinal studies that adjust for antipsychotic exposure indicate that antipsychotic treatment is unlikely to account for all of the excess volume changes in schizophrenia [47, 48].

Overall, the longitudinal evidence demonstrates regions of greater progressive grey matter volume loss, particularly in fronto-temporal regions, in schizophrenia relative to controls from early in the illness, and antipsychotic use may contribute to this. However, whilst further work is needed to determine the potential contribution of other factors to excess changes in schizophrenia, several large longitudinal studies to date indicate that there is excess grey matter loss in schizophrenia that is not entirely accounted for by antipsychotic use.

### Cortical thickness

Cortical thickness refers to the depth of the cortical grey matter in the orthogonal plane from the surface of the cortex, demarcated by the pia mater superficially and the white matter below. Regional variation in cortical thickness ranges from 1 mm (typically in Brodmann’s area 3) to 4.5 mm (generally Brodmann’s area 4) [64]. In 2018, van Erp et al. conducted a meta-analysis of cortical thickness and surface area abnormalities in schizophrenia. They collated data collected from 4474 individuals with schizophrenia and 5098 healthy volunteers. The schizophrenia individuals were found to have thinner cortices than healthy controls (right/left hemisphere: *d* = −0.52/−0.53 respectively) and in fronto-temporal regions (*d* < −0.40). Specifically, the largest negative effect sizes were in the fusiform gyri (right *d* = −0.54, left *d* = −0.49); superior temporal gyri (right *d* = −0.44, left *d* = −0.44); middle temporal gyri (right *d* = −0.38, left *d* = −0.44); inferior temporal gyri (right *d* = −0.44, left *d* = −0.45); left superior frontal gyrus (*d* = −0.43); right pars opercularis (*d* = −0.42) and insula cortex (right *d* = −0.41, left *d* = −0.41). With the exception of findings in the left superior frontal gyrus, most of the fronto-temporal differences remained statistically significant after controlling for global cortical thickness, which suggests lower cortical thickness in schizophrenia is particularly seen in fronto-temporal regions. Furthermore, there was a negative correlation between increasing age and bilateral temporal pole thickness, which was stronger in the schizophrenia group than in the healthy volunteers, suggesting an underlying process of progressive cortical thinning in schizophrenia consistent with the evidence for longitudinal grey matter volume loss, as outlined above [65].

As has been acknowledged elsewhere in this review, MRI findings can be influenced by multiple factors in addition to brain structure, and these need to be considered in their interpretation. In the case of cortical thickness, Weinberger & Radulescu (2021) outline that MRI findings suggestive of lower cortical thickness may, in fact, reflect increased myelin, which decreases T1 time and, in turn, the MRI measurement [66]. Thus, it is important to recognise that lower cortical thickness in schizophrenia may reflect alterations in other brain constituents rather than grey matter differences.

### Neurite orientation dispersion and density imaging

Neurite Orientation Dispersion and Density Imaging (NODDI) is a type of diffusion weighted MRI that can distinguish between microstructural components in grey and white matter. It can be applied to imaging of grey matter regions to extract measures such as the Neurite Density Index (NDI) and Orientation Dispersion Index (ODI). NDI characterises the density of neurites by modelling the intra-neurite space, whilst ODI models the space between axons to calculate the angular variation of neurites, cell membranes, somas and glial cells [67–69].

Since the method was first published in 2012 [67], we identified only one study that has used it to investigate grey matter microstructure in patients with chronic schizophrenia [70, 71]. Patients showed lower NDI relative to healthy controls in the temporal pole, anterior hippocampal gyrus and hippocampus [70]. Studies of patients with psychotic disorders including schizophrenia additionally report lower ODI relative to healthy controls in the anterior cingulate cortex and medial frontal gyrus [72] and lower NDI in lateral prefrontal cortex, superior/medial frontal gyrus, central sulci, superior temporal gyrus, and middle temporal gyrus [73]. However, given that these studies include some patients with affective psychosis, these findings must be interpreted with caution in relation to schizophrenia [73]. Finally, one study carried out in first episode psychosis patients, taking medication, that focussed on the hippocampus as the only region of interest, found no differences in ODI or NDI [74].

Together these studies suggest that schizophrenia may be associated with disrupted grey matter microstructures, but there are inconsistencies. Further studies are required to replicate these findings, and to investigate the potential contribution of confounding factors such as antipsychotic use. Notwithstanding these caveats, lower NDI could partly reflect lower levels of pre/postsynaptic elements. However, it is not a direct measure of synaptic elements, and changes could reflect alterations in other microstructural elements, such as lower density or atrophy of myelinated axons [75, 76].

### Summary of structural MRI findings and consideration of their implications

Meta-analyses demonstrate lower cortical and subcortical grey matter volumes and cortical thickness in patients with schizophrenia relative to healthy controls, with small to moderate effect sizes, although absolute volume differences are modest (~7% in fronto-temporal regions, and ~2% for whole brain grey matter [77]). Grey matter changes are progressive over time, from early in the course of the illness. Furthermore, grey matter loss in clinically at-risk groups is linked to subsequent transition to psychosis [43]. There are a number of potentially confounding factors in addition to antipsychotic treatment, including higher rates of substance misuse and higher incidence of physical and psychiatric comorbidity in patients with schizophrenia than in healthy controls, which could putatively contribute to findings [50], although we were not able to find evidence to support their effects in the meta-analyses so it remains unclear how much they might contribute to findings, if at all. There are also important methodological considerations in addition to those already raised. In particular, people with schizophrenia show increased motion in scanners compared to healthy control subjects [78]. Whilst a variety of methods have been used to correct for motion, small differences in motion are difficult to fully correct and could contribute to case-control differences [79, 80]. Additionally, around 80% of the studies in the meta-analyses we have considered used 1.5 Tesla (T) scanners [41], which may underestimate volume differences compared to 3 T scanners [81].

Another important consideration for interpreting findings is whether gender differences may impact the conclusions. While studies typically control for the male:female ratio between patients and controls, most also include more male participants (e.g ~66% male in Kuo and Pogue-Geile, 2019, and 67.4% male in Brugger & Howes, 2017) [41, 42]. On average males have greater total brain volume than females, with regional differences in both volume and density [82]. Several studies also suggest that there may be effects of gender on grey matter differences in schizophrenia in some regions but not others [83], highlighting the importance of investigating and controlling for gender effects in neuroimaging studies.

Notwithstanding these issues, what do these MRI measures reflect at the cellular level?

**Fig. 4:**
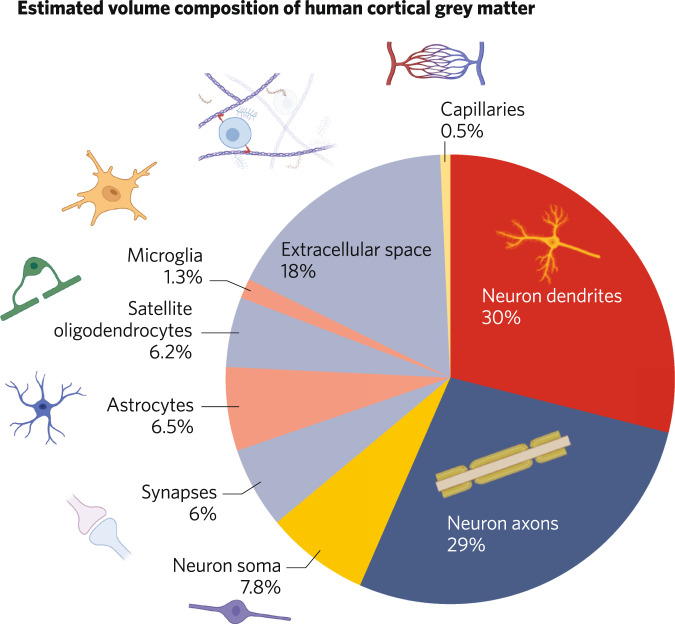
Illustrating the estimated relative composition by volume of 1 mm^3^ of human cortical grey matter, showing that the major constituents are axons and dendrites, with synapses contributing relatively little. Adapted with permission from Bennett 2011.

Given the huge number of human MRI studies that measure grey matter parameters, there has been surprisingly little work to investigate what cellular constituents make up MRI grey matter measures [84]. Bennett has provided some estimates for the constituents of 1 mm^3^ of human cortical grey matter derived from a review of the literature, summarised in Fig. 4 [15]. This highlights that axons and dendrites make up the largest proportion of grey matter, and synapses make up a relatively small component by volume (around 6%). An important caveat is that these are estimates for average grey matter, and may vary across the brain. Moreover, human data for some structures, such as the volume of axon collaterals, are not available so were estimated from data from other species such as cat [15].

**Fig. 5:**
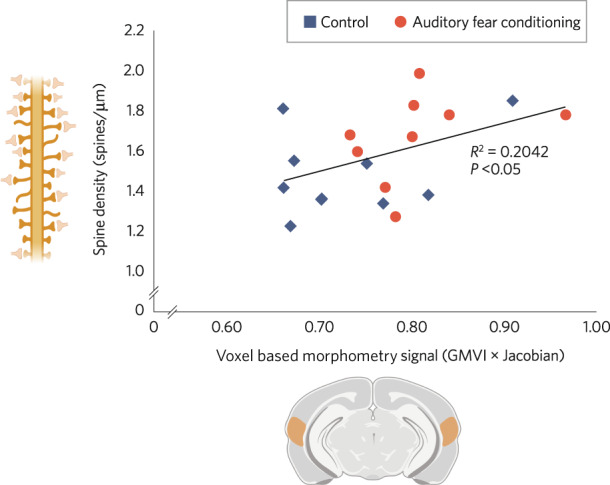
The relationship between spine density and grey matter volume in a rodent experiment. Scatter plot showing a significant correlation between spine density values with the voxel-based morphometry signal (Grey Matter Voxel Intensity (GMVI) x Jacobian) in the auditory cortex of mice in a fear-conditioning experiment (*n* = 9 per group *p* = 0.027, Pearson’s correlation *R*^2^ = 0.2042, blue = control group, red = auditory fear-conditioned group). Adapted with permission from Keifer et al., 2015.

A related question is what changes in cellular structure can lead to grey matter volume change. A number of studies have investigated this using animal models. Kassem et al. used restraint stress, which is known to cause brain structural changes, to investigate this using MRI in mice [85]. They found stress resulted in grey matter volume loss of the order of 10–15% in some, but not all, brain regions investigated. The grey matter volume loss occurred in the absence of loss of cells, but with reductions in synaptic spine density and dendritic volume. Moreover, change in grey matter volume was directly correlated with change in dendritic volume. Another study used a learning paradigm, auditory fear conditioning, in mice to show this was associated with both increases in spine density and greater grey matter volumes in the same brain regions [86]. Moreover, these were directly correlated (Fig. 5), but there was no significant relationship between nuclei density and the VBM signal in the same region. The R^2^ was ~0.2, indicating that ~20% of the variance in the MRI signal was related to differences in synaptic spine density. Similar findings have been seen in other species as well. For example, a non-human primate study using a stroke model [87] showed lower grey matter volumes on MRI and decreased levels of synaptic markers in the same region in the absence of cell loss.

These studies were cross-sectional so do not show relationships with change in MRI signal. To address this, Asan et al. used a longitudinal neuroimaging approach to investigate age related changes in grey matter volume and cellular constituents [84]. Mice received structural MR imaging and two-photon in vivo imaging (2pii) at three timepoints over 12 weeks. 2pii was employed to measure a range of parameters including cellular shrinkage (or expansion) over time; nucleus count and cell density; distances between cell nuclei (thus cell clustering); and mean nuclei volume (as a marker for transcriptional activity). Interestingly, they found grey matter reductions over time, but that changes in cell number were not correlated with grey matter volume across imaging volumes capturing all cortical layers, as is typically the case in human imaging. Unfortunately, they do not report data for synaptic density or volumes.

One important methodological consideration is that animals are typically anaesthetised during MRI studies, in contrast to the human studies, and physically restrained in other studies. Both factors reduce the potential for movement artefacts that may affect human imaging. We were unable to find studies that quantified both pre- and postsynaptic elements as well as grey matter volumes in the same animal. Future studies that do this would be useful to fully quantify the relationship between grey matter volume measures and synaptic measures. Nevertheless, while there is limited translational evidence, taken together, the findings from the studies we have discussed show that grey matter volume is partly related to synaptic spine density, and changes can occur in grey matter volumes without changes in cell number. Of course, this merely indicates it is possible that lower grey matter volumes in schizophrenia could be related to synaptic loss, but does not provide evidence that this is the case [84].

Indeed, a number of researchers, including Lerch et al. [88] and Weinberger & Radulescu [66], have called for caution before drawing molecular and cellular conclusions from in vivo MRI imaging data. MRI findings reflect the local magnetic properties of the tissue being imaged [66]. As such, structural MRI findings can be influenced by alterations in water content, tissue perfusion, cholesterol levels, and imperceptible head motion, any of which may be different between groups [66]. Although dimensional MRI findings indicative of altered grey matter volume are often interpreted in terms of the synaptic hypothesis, it is important to recognise that these other factors could account for alterations, and the need for more specific measures of synapses in patient studies.

### Studies of neuronal metabolism

Glucose utilisation is a marker of cerebral metabolism that can be studied directly with PET, using the radiolabelled glucose analogue 18-flurodeoxyglucose (FDG). FDG is taken up by neurones and enters the first step of the metabolic pathway. However, it cannot be utilised further, and so accumulates, providing a marker of glucose utilisation, where neuronal energy demand is proportional to the FDG signal [89].

Thirty-six case-control FDG brain studies in schizophrenia were recently meta-analysed [90]. Moderate to large reductions in resting, but not task-related, FDG uptake were found in a sample of over 1300 subjects (642 schizophrenia patients compared to 693 controls). Reductions were found in the frontal lobe, both in terms of absolute FDG uptake (*g* = −0.66) and uptake relative to uptake in the remaining cortex (*g* = −0.44) [90]. There was an effect of illness duration on absolute resting frontal FDG uptake, which was significantly lower in chronic schizophrenia than controls, but there was no difference between first episode psychosis and controls. There was also an effect of medication status on resting FDG uptake, with subgroup analyses showing that frontal uptake (relative to the remaining cortex) was significantly lower in medicated/mixed than drug-naïve/drug-free cohorts (*p* < 0.01). However, most (5/6) cohorts of medication-naïve patients were experiencing their first episode of psychosis; thus, it is not possible to disentangle potential effects of antipsychotic use from stage of illness [90]. Longitudinal studies, investigating temporal changes in resting frontal FDG uptake during the course of illness and treatment, are required to examine this further.

The neurometabolite N-acetylaspartate (NAA) is also thought to reflect neuronal metabolic function [91], and NAA levels have been shown to correlate positively with FDG uptake in the grey matter of healthy and cognitively impaired volunteers [92], as well as in the posterior cingulate cortex in Alzheimer’s disease [93]. A recent meta-analysis found lower NAA levels in people with chronic schizophrenia, in fronto-temporal and parietal regions (*g* = −0.52 to −0.25), and, to a lesser degree, in first episode patients with a potential effect of treatment [94] (see supplement for details), similar to the FDG PET findings.

What does this evidence of lower metabolic markers tell us about synaptic density? Neurotransmission is energy expensive, with two-fifths of all cortical ATP spent on synaptic neurotransmission [95], and so lower synaptic density could underlie case-control differences in regional glucose metabolism. Potentially supporting this interpretation, Chen et al. recently showed that, in patients with Alzheimer’s disease, resting FDG PET signal was positively correlated with UCB-J PET signal, a synaptic protein marker, in several regions, with comparable reductions in both measures relative to controls in the medial temporal lobe [96]. In addition, global resting FDG and UCB-J PET signals are also positively correlated in healthy volunteers (*r* > 0.47, *p* < 0.001) [97] and regional resting FDG uptake strongly correlates with synaptophysin expression in the baboon [98].

However, Chen et al. (2021) also found that, in Alzheimer’s disease, reductions in FDG signal exceeded reductions in UCB-J signal in the neocortex [96]. There are several potential explanations for this, such as a change in synaptic activity without a change in synapse number [99], or neocortical loss of non-neuronal cells, such as astrocytes, which also contribute significantly to whole brain FDG uptake [100]. This highlights that FDG uptake is not specific to synaptic function and may be influenced by activity in other neuronal and non-neuronal structures. Moreover, relationships between FDG uptake and synaptic density in Alzheimer’s disease or healthy volunteers may not necessarily translate to schizophrenia. Similar considerations apply to the interpretation of NAA levels (see Supplementary Information).

### Imaging synaptic terminal markers

Synaptic Vesicle Protein 2 A (SV2A) is a protein ubiquitously expressed in synaptic terminals throughout the brain [101]. Studies have shown it is involved in calcium-dependent neurotransmitter release in both inhibitory and excitatory terminals [101–103]. Over the last decade, a number of PET tracers for SV2A have been developed, including [11 C] radiolabelled ligands such as [11 C]UCB-J and [11 C]UCB-A, and the [18 F] radiolabelled tracers [18 F]-UCB-H and [18 F]SynVesT-1 [104–107]. Of these, [11 C]UCB-J has been the most extensively used to date [108–112]. It shows favourable kinetics for PET imaging, and human blocking studies show it selectively binds to SV2A [113, 114]. It has also been shown to have good test-retest reliability and tracer binding has been shown to remain stable at rest and during task conditions, indicating that the signal is not activity dependent [114–116]. SV2A measures have been shown to directly correlate with those of synaptophysin, the most widely used post-mortem marker for synapses [114].

**Fig. 6:**
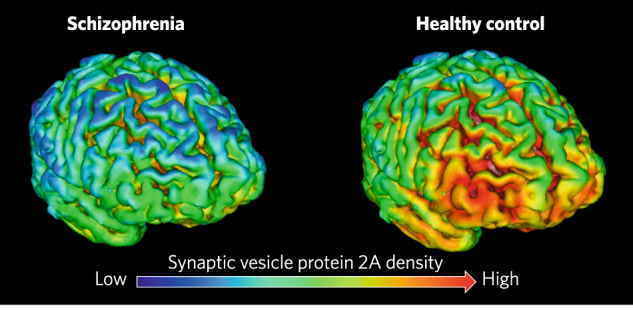
Whole brain maps of the mean [11 C]UCB-J volume of distribution (*V*_T_) in schizophrenia (left) and healthy control (right) groups, indicating lower levels of the synaptic vesicle protein 2 A (SV2A) marker in schizophrenia particularly in fronto-temporal regions, consistent with lower synaptic density in schizophrenia (based on data from Onwordi et al. 2020). Low [11 C]UCB-J uptake (dark blue) corresponds to *V*_T_ values in the 10–15 ml/cm^3^ range, high [11 C]UCB-J (orange-red) corresponds to *V*_T_ values in the 20–25 ml/cm^3^ range. Image credit: Onwordi and Howes (2022).

To date, two studies have been published using [11 C]UCB-J to compare patients with chronic schizophrenia with matched controls (see Table 2) [117, 118]. In the first study, the authors selected three primary regions of interest based on findings from a meta-analysis of post-mortem studies that found lower synaptic protein levels in schizophrenia with moderate-large effect sizes in frontal and anterior cingulate cortices and hippocampus [6]. They found significantly lower [11 C]UCB-J uptake in frontal and anterior cingulate cortex with large effect sizes (*d* = 0.8 to 1.0), and a trend for significantly lower levels in the hippocampus (*d* = 0.6, *p* = 0.09). Exploratory analyses indicated significantly lower binding in some, but not all, additional brain regions examined, including dorsolateral prefrontal and temporal cortices and subcortical regions including the thalamus and amygdala. Figure 6 shows the spatial distribution of mean uptake in the patient and control groups, highlighting that differences seem to be particularly pronounced in fronto-temporal regions. The second study also found significantly lower [11 C]-UCB-J uptake in frontal cortex, including the anterior cingulate cortex, as well as in the hippocampus, occipital, parietal and temporal cortices of patients with schizophrenia.

**Table 2:** [11 C]UCB-J imaging studies in patients with schizophrenia, summarising sample details.

Studies	Sample size		Mean age ± SEM		Mean illness duration (SCZ)
	SCZ	CTR	SCZ	CTR	
Onwordi et al. (2020)	19 (3 F)	18 (3 F)	41.5 ± 2.7	38.7 ± 3.1	17.4 years
Radhakrishnan et al. (2021)	13 (3 F)	15 (3 F)	40.52 ± 3.1	40.77 ± 2.9	17.3 years

*CTR* control subjects, *SCZ* patients with schizophrenia, *F* female.

Both studies included patients taking antipsychotic treatment. However, neither study found a relationship between cumulative antipsychotic dose and [11 C]UCB-J uptake [117, 118]. Moreover, Onwordi et al. tested if sub-chronic haloperidol or olanzapine exposure affected SV2A markers in rodents, showing that binding of [3H]UCB-J and levels of other SV2A markers were unaffected by the antipsychotic administration [117]. Thus, the data to date suggest that antipsychotic treatment is not confounding these findings. Nonetheless, confirming whether patients with schizophrenia who are not taking any medication have lower SV2A levels remains an outstanding question.

## Discussion

Our review summarises findings from meta-analyses of brain imaging findings in over 50,000 subjects (including over 28,000 patients), identifying alterations in a number of structural, synaptic protein and metabolic markers relevant to understanding whether there are synaptic alterations in schizophrenia (Table 3). This shows that, in comparison to control subjects, grey matter volumes are lower in patients with chronic schizophrenia, in first episode patients and, to lesser degrees, in people with risk factors for schizophrenia. These differences are typically most pronounced in the frontal and temporal cortices both in chronic schizophrenia and first episode patients and, to some extent, in clinically high-risk individuals who go on to develop psychosis, who have lower grey matter volumes of the superior temporal gyrus. The grey matter findings in schizophrenia are based on meta-analyses of over 600 studies and more than 23,000 patients. It would take a large number of negative findings for these structural findings to be nullified, meaning that we can be confident that schizophrenia is associated with lower grey matter volumes in a number of cortical and subcortical brain regions, particularly in frontal and temporal cortical regions. Longitudinal structural MRI findings also show that grey matter volume loss is accelerated in schizophrenia in comparison to healthy controls, and that this is at least partially independent of antipsychotic exposure, although more studies are warranted to further test this (see supplement for further discussion).

**Table 3:** Summary of imaging findings in schizophrenia and people at risk for schizophrenia, from the literature discussed in this review, and key considerations for understanding if there are synaptic density changes in the disorder.*

Modality and measure	Main findings in genetically (GHR) and clinically high risk (CHR)	Main findings in first episode psychosis (FEP)	Main findings in chronic schizophrenia	Longitudinal findings	Effects of antipsychotic medication	Limitations
Structural MRI: gyrification index	No meta-analysis: studies generally support ↑	No meta-analysis: studies generally support ↑	Meta-analysis shows ↓ globally (systematic review generally supports this, especially in older patients)	No meta-analysis: some studies show faster rate of decrease in schizophrenia	No meta-analysis: limited indirect evidence for widespread ↓ from longitudinal studies	Limited number of studies, particularly in early illness and of longitudinal change. No studies yet link GI to synaptic markers
Structural MRI: cortical grey matter (GM) volume	GHR: ↓ thalamus ↑ ventricles ↔ accumbens ↔ amygdala ↔ hippocampus ↔ pallidum ↔ putamen CHR (who transition to psychosis relative to those who do not transition): ↓ anterior cingulate ↓ superior temporal gyrus	↓ amygdala ↓ anterior cingulate ↓ frontal lobe ↓ hippocampus ↓ thalamus ↓ temporal lobe ↑ ventricles ↔ caudate ↔ putamen	↓ frontal lobe ↓ fusiform ↓ hippocampus ↓ insula (L) ↓ prefrontal lobe ↓ superior temporal gyrus ↓ temporal lobe ↓ total brain volume ↑ ventricles	Greater volume loss of: total cortical GM, superior temporal gyrus, Heschl gyrus (L), planum temporale (L), frontal cortex, temporal cortex, thalamus, caudate nucleus, insula, periventricular area, putamen, cingulate cortices Rate of GM volume loss most pronounced early in illness	Drug free patients: ↓ fusiform gyrus (L) ↓ inferior frontal gyrus (L) ↓ superior temporal gyrus (R) ↓ supramarginal gyrus (L) ↑ lingual gyrus (R) ↑ superior frontal gyrus (R) ↑ progressive cortical GM volume loss associated with ↑ daily antipsychotic intake in patients taking at least one first-generation APD *vs*. those taking second-generation only	Not specific to synapses, and could include differences in glia, neuronal soma volumes, dendrites and other structural components
Structural MRI: cortical grey matter density	n/a	n/a	↓ insular cortex (bilateral) ↓ anterior cingulate ↓ parahippocampal gyrus (L) ↓ middle frontal gyrus ↓ postcentral gyrus ↓ thalamus ↑ putamen ↑ caudate (R)	n/a	n/a	Not specific to synapses, and could include difference in glia, neuronal soma volumes, dendrites and other components
Structural MRI: cortical thickness	n/a	n/a	↓ hemispheric cortical thickness bilaterally ↓ fusiform (bilateral) ↓ superior, middle and inferior temporal gyri (bilateral) ↓ superior frontal gyrus (L) ↓ pars opercularis (R) ↓ insula (bilateral)	Stronger negative correlation between increasing age and bilateral temporal pole thickness in schizophrenia than in controls	↓ frontal lobe ↓ parietal lobe ↓ temporal lobe ↓ insula ↓ isthmus of cingulate gyrus (in medicated *vs* unmedicated and *vs* controls)	Not specific to synapses, and could include difference in glia, neuronal soma volumes, dendrites and other components
Neurite orientation dispersion and density imaging	n/a	↔ hippocampus n/a for other ROIs	↓ temporal pole ↓ anterior hippocampal gyrus ↓ hippocampus	n/a	n/a	Not specific to synapses and could include differences in myelination of axons
FDG PET: glucose utilisation	n/a	↓ frontal cortex in chronic schizophrenia vs. FEP ↔ frontal cortex in FEP vs. controls	↓ frontal cortex	n/a	↓ frontal cortex in medicated/mixed patient vs. drug-naïve/drug-free patient cohorts	Changes in FDG uptake may represent changes in neuronal activity (not density) Significant FDG uptake by non-neuronal cells
MRS studies of N-acetylaspartate (NAA) levels	↓ hippocampus	↓ frontal cortex ↓ anterior cingulate ↓ thalamus	↓ hippocampus ↓ frontal cortex ↓ temporal cortex ↓ parietal cortex	n/a	In unmedicated FEP: ↓ frontal lobe ↓ anterior cingulate cortex ↓ dorsolateral prefrontal region ↓ frontal white matter ↓ thalamus	Expression of NAA is not specific to synapses
[11 C]UCB-J PET: Synaptic Vesticle Protein 2 A (SV2A) levels	n/a	n/a	↓ frontal lobe ↓ anterior cingulate ↓ hippocampus and potentially some other cortical regions	n/a	n/a	Does not measure postsynaptic elements

*MRI* magnetic resonance imaging, *GM* grey matter, *FDG* fluorodeoxyglucose, *PET* positron emission tomography, *MRS* magnetic resonance spectroscopy, *NAA* N-acetylaspartate, *SV2A* synaptic vesticle protein 2A, *GHR* genetic high risk, *CHR* clinical high risk, *FEP* first episode psychosis,  *ROI* region of interest, *APD* antipsychotic drugs, *R* right, *L* left, ↓ lower in patient group relative to controls, ↑ higher in patient group relative to controls, ↔ no significant difference between patient and controls groups.

*Findings are in patients versus controls, unless otherwise stated.

Gyrification is lower in studies of older patients with chronic illness, but there are inconsistent findings in first episode patients, indicating that there is a need for more, ideally longitudinal, studies early in the course of illness to determine the trajectory of change. Measures of brain metabolism are also lower in schizophrenia, particularly in frontal regions, but effects are most marked in patients with chronic illness.

Finally, two recent studies using [11 C]UCB-J PET find evidence for lower levels of the synaptic terminal protein SV2A, in frontal and temporal cortices of patients with schizophrenia. The SV2A PET imaging in schizophrenia thus extends post-mortem findings of lower protein and mRNA levels of synaptophysin and other presynaptic markers in samples from patients in these regions [6, 117, 118].

### Strengths and limitations

We were able to identify meta-analyses synthesising data from large numbers of studies for many of the measures. However, for some measures meta-analyses were not available (see Table 3), generally because there were insufficient studies. This was notably the case for gyrification measures, cortical thickness and grey matter density measures, where there were no meta-analyses of studies in first episode patients or high-risk groups, and for SV2A studies at all stages of schizophrenia. This limits the conclusions that can be drawn for these groups and measures. This was also the case for longitudinal studies in general. This limits the conclusions that can be drawn about whether there are progressive changes in many measures, and highlights the need for more longitudinal studies in schizophrenia.

#### What do the imaging findings say about whether there is lower synaptic density in schizophrenia?

The preclinical studies that we reviewed show that interventions that alter ex vivo synaptic markers are also associated with changes in MRI measures. This indicates that the MRI findings in schizophrenia could reflect lower synaptic density. However, only a few studies have investigated the relationship between MRI imaging signals and synaptic measures, and these have predominantly been conducted in rodent models. There is a need for further studies to determine if these rodent findings can be replicated, and for studies in humans (such as post-mortem studies combining MRI imaging and synaptic measures). Furthermore, as shown in Fig. 4, over 50% of grey matter volume is estimated to comprise dendrites and axons, 18% is extracellular space, and the remaining volume includes neuronal soma, synapses, astrocytes, oligodendrocytes and glia. Given that synapses are estimated to comprise only ~6% of total grey matter volume, and grey matter volume differences in schizophrenia are similar in magnitude (~5–11% in some fronto-temporal regions [77]) and it is implausible all synapses are lost in schizophrenia, synaptic loss alone is unlikely to explain all of the grey matter and cortical thickness differences seen in schizophrenia. Moreover, whilst post-mortem studies do not generally find lower density of neurons or other cells in brain regions of interest in schizophrenia, there are reports of lower number and volume of neurons in some regions [61, 119–124], which could contribute to the grey matter MRI differences. Nevertheless, although lower synaptic density is unlikely to account for all of the grey matter differences in schizophrenia, this does not preclude synapse loss being a driver of changes in other constituents, such as dendritic volume, that might also contribute to volume loss. As discussed earlier, NAA and FDG measures are also not specific to synapses. Thus, whilst the alterations in these and the structural measures are consistent with lower levels of synapses, they cannot be taken as establishing this. In this respect, imaging of SV2A provides the most specific measure of synaptic density currently available.

### Gaps in evidence and the interpretation of SV2A findings in terms of lower synaptic density

Box 1 summarises a number of the key outstanding areas for further work identified by our review of imaging findings in schizophrenia. The gaps in evidence and outstanding questions are considered further in this section before we then consider the potential mechanism that could underlie lower synaptic terminal density. The fact that lower [11 C]UCB-J levels have been reported in two independent cohorts of patients provides some confidence that there are lower SV2A levels in schizophrenia. Nevertheless, it will be important for further studies to confirm findings in additional cohorts to determine the generalisability of findings, and to include antipsychotic free patients (Box 1). Both [11 C]UCB-J studies to date were of patients with chronic schizophrenia. It is thus unclear at what stage of illness these deficits arise and whether differences in SV2A reflect earlier loss of synapses, the effects of disease progression or potentially both. The evidence for longitudinal changes in grey matter volumes from first episode suggests there may be early and progressive changes in neuropil, which might include synaptic loss, in schizophrenia, but multimodal studies earlier in the course of illness are required to determine the degree to which synaptic differences contribute to this (Box 1).

As discussed earlier, SV2A is a presynaptic vesicular protein expressed in synaptic terminals. However, whilst the SV2A findings could reflect a selective loss of synaptic terminals, or even just vesicle proteins, in schizophrenia, when they are taken with the post-mortem evidence for lower levels of pre- and postsynaptic markers in the same regions in schizophrenia, the most likely interpretation is that they reflect lower synaptic density in the disorder. This supports the synaptic hypothesis of schizophrenia [2, 3, 5]. Nevertheless, confirming that there is loss of postsynaptic elements in vivo as well will require the development of new PET tracers or other imaging approaches. In the meantime, further post-mortem studies to investigate the levels of synaptic vesicle proteins, the number of SV2A molecules per vesicle and the number of vesicles per terminal in schizophrenia, would be useful to aid interpretation of the in vivo findings.

SV2A is expressed at both excitatory and inhibitory synapses [101]. Whilst this means it is a good marker of total synaptic terminal density, a limitation of [11 C]UCB-J imaging is that it provides little information about what type of synapses may be affected in schizophrenia. Recent work by Onwordi et al. combined [11 C]UCB-J with MRS of glutamate levels to investigate the relationship between the two measures [125]. The study found that glutamate levels were correlated with the SV2A measure in the anterior cingulate cortex of control subjects, but not in the patient group, which, taken together with lower SV2A levels in this region, suggests excitatory synaptic terminals may be particularly affected in schizophrenia [125]. However, while the highest brain glutamate concentrations are found in glutamatergic nerve terminals, the glutamate MRS signal also reflects glial and extracellular glutamate levels, potentially limiting the interpretation of these findings [126].

As also discussed earlier, the structural MRI differences in schizophrenia could reflect alterations in other neuropil components, such as dendrites, unmyelinated axons, glial cells and not just synapses. They could also reflect differences in other grey matter constituents, including cell body number and volume, in addition to neuropil. Supporting this, neither [11 C]UCB-J PET study found SV2A levels were related to grey matter volumes. However, given that, at most, synaptic differences will only account for a proportion of structural brain differences in the same regions, the samples for these studies were likely under-powered to detect relationships. Moreover, neither study included other structural or metabolic measures, such as cortical thickness or FDG PET. Thus, larger studies that investigate the relationships between the structural and metabolic measures are needed to understand the degree to which synaptic changes could underlie the structural and metabolic brain changes we review in schizophrenia (Box 1).

Some questions remain about the effects of antipsychotic medication. While neither UCB-J study found effects of cumulative antipsychotic dose on SV2A levels, this does not exclude an effect of antipsychotic drugs. Thus, studies of SV2A levels in antipsychotic untreated patients are needed to rule this out. In addition to this, use of other drugs such as cannabis or cocaine may influence findings [127, 128]. While both UCB-J studies in schizophrenia excluded participants who tested positive for drugs of abuse, cohorts of patients with schizophrenia typically have higher frequency of past drug use, which could confound findings [129]. However, recent work shows SV2A levels are more likely to reflect recent use rather than be correlated with past substance use history, although this has only been tested for cocaine to date [127]. Whilst this suggests that past substance use is not likely to be a major confound, further work is warranted to determine if this holds for other substances in addition to cocaine.

Finally, grey matter loss, cortical thinning and lower SV2A levels have been found in other neuropsychiatric disorders, such as major depression, substance use disorders, post-traumatic stress disorder, and others [111, 127, 128]. This raises a number of questions, including how specific the alterations we have considered are to schizophrenia, and whether the same mechanism underlies alterations across disorders. Key differentiators may be the underlying mechanisms and the circuits involved.

What mechanism could underlie synaptic loss? Immune-mediated excessive synaptic pruning has been proposed as one possibility [130, 131]. Supporting this, numerous post-mortem studies have demonstrated increased density of microglial cells (with a hypertrophic morphology indicating an activated phenotype) in the brains of schizophrenia patients compared with healthy controls, particularly in the frontal and temporal lobes [130, 132–134]. Microglial cells are the immune cells of the central nervous system, which play a major role in synaptic pruning and phagocytosis of synaptic material in the brain [135, 136]. In vitro studies further show increased engulfment of synaptosomes by microglia cultured from patients with schizophrenia in comparison to controls [13], suggesting a schizophrenia genetic background may contribute to excessive synaptic pruning in patients. Other risk factors for schizophrenia may also lead to microglial activation [137]. For example, juvenile stress exposure in rodents has been shown to result in increased microglial pruning and synaptic loss, particularly in the prefrontal cortex and the hippocampus [138–146]. Adolescent stress is additionally considered a risk factor for developing schizophrenia, potentially implicating the same pruning mechanism [147]. Thus, aberrant microglial activation is a potential mechanism that could account for lower synaptic density in schizophrenia, and disrupt cortical function (see [5] for a further discussion of this). However, there is inconsistency within the in vivo findings on microglial markers in schizophrenia, potentially due to methodological factors [148], and the link between microglial mediated pruning and brain imaging changes in vivo remains to be established. Further studies are also required to investigate the effects of stress on SV2A levels, and to relate this to schizophrenia pathophysiology. Notwithstanding these caveats, this suggests potential targets for interventions [137].

Box 1: Key next steps

- Further longitudinal studies of grey matter volumes from the first episode of schizophrenia controlling for antipsychotic use
- Additional studies of monozygotic and dizygotic relatives of patients with schizophrenia to determine the shared genetic and environmental components to grey matter differences in schizophrenia
- Further studies in first episode patients and of longitudinal changes in gyrification and cortical thickness
- SV2A imaging in additional cohorts of patients with schizophrenia to test generalisability of findings
- Investigation of the relationship between SV2A and structural and functional imaging measures in schizophrenia
- SV2A imaging in first episode schizophrenia to determine if differences are present early in the course of illness

## Conclusions

Neuroimaging techniques have been widely used to investigate the pathophysiology of schizophrenia, providing evidence for a range of structural and metabolic differences in schizophrenia, including lower grey matter volumes, cortical thickness, gyrification and NAA and FDG levels with moderate effect sizes in chronic schizophrenia in cortical regions, particularly in fronto-temporal areas. Effects are also seen in first episode schizophrenia, with the possible exception of gyrification. Moreover, evidence indicates grey matter changes are progressive from the first episode of illness. These findings are consistent with, but do not prove, lower synaptic density in the disorder. Moreover, it is clear that synaptic differences alone cannot account for the magnitude of the grey matter changes. Nevertheless, recent findings using a PET tracer specific for a presynaptic protein (SV2A) provide in vivo evidence for lower synaptic terminal density in fronto-temporal regions in the disorder, consistent with the post-mortem evidence for lower levels of pre- and postsynaptic markers in schizophrenia. Questions remain regarding whether synaptic alterations are present at illness onset or develop later, and the relationship between them and the other neuroimaging findings we have reviewed.

## Supplementary information

Supplement
