# Supplementary material for: Neuroimaging in schizophrenia: an overview of findings and their implications for synaptic changes
Source: Neuropsychopharmacology. 2022 Sep 2;48(1):151–67. doi: 10.1038/s41386-022-01426-x (PMC9700830; doi:10.1038/s41386-022-01426-x)
Supplement: Supplementary file 1 — Supplement [file 41386_2022_1426_MOESM1_ESM.docx]

Supplementary materials for ‘Neuroimaging in schizophrenia: an overview of findings and their implications for synaptic changes’

Expanded discussion of antipsychotic use and volumetric findings

Some preclinical evidence suggests that exposure to antipsychotic medication may contribute to grey matter change [1], thus, investigating whether this may be the case in schizophrenia remains an important question. One MRI meta-analysis addressed this by analysing data from 1046 schizophrenia patients and 780 healthy controls including studies with a median duration follow-up of 72.4 weeks. This replicated findings of progressive grey matter volume reductions in schizophrenia, but found that longitudinal decrease in grey matter volume was associated with cumulative exposure to antipsychotic treatments [2]. Furthermore, in 2015, a meta-analysis and meta-regression of longitudinal MRI studies by Vita et al. observed a similar relationship between total cortical grey matter volume loss and cumulative antipsychotic intake in data from 1155 patients with schizophrenia and 911 healthy controls. Interestingly, subgroup analysis found that the effects of antipsychotic use was class-dependent: more progressive cortical grey matter volume loss was associated with higher mean daily antipsychotic intake in patients treated with at least one first-generation antipsychotic compared patients treated with second-generation antipsychotics alone [3].

These meta-analyses thus provide evidence that antipsychotic treatment could contribute to grey matter volume changes in schizophrenia, but are based on cross-sectional associations. Two large longitudinal studies have investigated this issue further, by adjusting for antipsychotic exposure in their analyses. Both found that the excess reduction in grey matter volumes in schizophrenia relative to controls was not wholly accounted for by antipsychotic exposure [4, 5]. Whilst this indicates antipsychotic exposure does not explain all of the grey matter volume loss in schizophrenia, further investigation is required to test the potential role of antipsychotics in these changes. It should also be noted that the degree of antipsychotic use may be confounded by illness severity as more severely ill patients are more likely to be to receive treatment and at higher doses.

Expanded discussion of N-acetylaspartate differences in schizophrenia

N-acetylaspartate (NAA) is one of the brain’s most abundant metabolites, levels of which are thought to reflect neuronal metabolic function [6]. NAA can be measured *in vivo* using magnetic resonance spectroscopy (MRS). A recent meta-analysis of 182 MRS studies (comprising 2339 schizophrenia patients and 2056 controls) found lower NAA levels in people with chronic schizophrenia, in hippocampal, frontal, temporal and parietal regions (*g =* –0.52 to –0.25) [7]. There were smaller, but still significant, reductions in NAA levels in first episode psychosis (in the frontal lobe, anterior cingulate cortex and thalamus) and those at high risk of psychosis (in the hippocampus) relative to controls. Interestingly, a sub-analysis of studies of unmedicated first episode psychosis patients did not find significant differences in NAA levels compared with controls, although there were trends for lower NAA levels in the untreated patients relative to controls in frontal lobe, anterior cingulate cortex, the dorsolateral prefrontal region, frontal white matter and thalamus. However, given that only a subset of studies in first episode patients were included here (e.g. 15/37 in frontal lobe, 5/10 in thalamus), and that frontal and thalamic sub-analyses were of the same direction as analyses of all first episode patients, the unmedicated analyses may reflect true but underpowered signal.

NAA levels are positively correlated with UCB-J PET signal in hippocampus and anterior cingulate in healthy controls [107], supporting a link between NAA levels and synaptic measures. However, there is a paucity of preclinical work investigating the relationship between NAA levels and synaptic and other cellular markers. Moreover, myelin and oligodendrocytes contribute to brain NAA levels [108]. Thus, alterations in NAA may not reflect synaptic changes.

Search terms used to identify key publications:

PubMed search 03-Feb -2022:

((Gray OR Grey) matter) AND (Schizophreni*AND (meta-analy* OR systematic OR Cochrane) = 340 results

((Gray OR Grey) matter) AND (Schizophreni*) AND (meta-analy* OR systematic OR Cochrane) AND (Gene*) 156 results

((Gray OR Grey) matter) AND (Schizophreni*) AND (meta-analy* OR systematic OR Cochrane) AND ([Clinical OR ultra] high-risk) 191 results

‘(cort* thickness) AND (Schizophreni*) AND (meta-analy* OR systematic OR Cochrane)’ = 72 results

(MRI OR magnetic resonance imaging) AND (schizophren* OR psycho*) AND (systematic review OR meta-analysis)

(structural neuroimaging) AND (schizophren* OR psycho*) AND (systematic review OR meta-analysis)

(gyrification OR gyrification index) AND (schizophren* OR psycho*) AND (review OR systematic review OR meta-analysis)

(fluorodeoxyglucose OR FDG OR cerebral metabol*) AND (schizophren* OR psycho*) AND (systematic review OR meta-analysis)

(NAA OR N-acetyl-aspartate) AND (MRS OR (magnetic resonance spectroscopy)) AND (schizophren* OR psycho*) AND (systematic review OR meta-analysis)

((NODDI) OR (neurite orientation dispersion and density imaging)) AND (schizophren* OR psycho*)

((synaptic vesicle protein 2A) OR (SV2A) OR (UCB-J)) AND (schizophren* OR psycho*)

Supplementary references:

1. Konopaske, G.T., et al., *Effect of chronic exposure to antipsychotic medication on cell numbers in the parietal cortex of macaque monkeys.* Neuropsychopharmacology, 2007. **32**(6): p. 1216-1223.

2. Fusar-Poli, P., et al., *Progressive brain changes in schizophrenia related to antipsychotic treatment? A meta-analysis of longitudinal MRI studies.* Neuroscience & Biobehavioral Reviews, 2013. **37**(8): p. 1680-1691.

3. Vita, A., et al., *The effect of antipsychotic treatment on cortical gray matter changes in schizophrenia: does the class matter? A meta-analysis and meta-regression of longitudinal magnetic resonance imaging studies.* Biological Psychiatry, 2015. **78**(6): p. 403-412.

4. Ho, B.-C., et al., *Long-term antipsychotic treatment and brain volumes: a longitudinal study of first-episode schizophrenia.* Archives of general psychiatry, 2011. **68**(2): p. 128-137.

5. Cahn, W., et al., *Brain volume changes in first-episode schizophrenia: a 1-year follow-up study.* Archives of general psychiatry, 2002. **59**(11): p. 1002-1010.

6. Moffett, J.R., et al., *N-Acetylaspartate in the CNS: from neurodiagnostics to neurobiology.* Progress in neurobiology, 2007. **81**(2): p. 89-131.

7. Whitehurst, T.S., et al., *Proton Magnetic Resonance Spectroscopy of N-acetyl Aspartate in Chronic Schizophrenia, First Episode of Psychosis and High-Risk of Psychosis: A Systematic Review and Meta-Analysis.* Neurosci Biobehav Rev, 2020. **119**: p. 255-267.
